# The Effectiveness of a Specific Exercise Program in Alleviating Work-Related Neck and Upper Back Pain and Improving Mood State in Various Occupational Populations—A Randomized Controlled Trial

**DOI:** 10.3390/medicina60122002

**Published:** 2024-12-04

**Authors:** Anastasia Beneka, Paraskevi Sakellari, Katerina Daskalaki, Paraskevi Malliou, Theodoros Konstantinidis

**Affiliations:** 1 Department of Physical Education and Sport Science, Democritus University of Thrace, 69100 Komotini, Greece; ampeneka@phyed.duth.gr (A.B.); psakella@phyed.duth.gr (P.S.); adaskala@phyed.duth.gr (K.D.); pmalliou@phyed.duth.gr (P.M.); 2 Department of Medicine, Democritus University of Thrace, 68100 Alexandroupolis, Greece

**Keywords:** musculoskeletal disorders, office employees, healthcare employees, neck pain, FS, VAS

## Abstract

*Background and Objectives:* The COVID-19 pandemic brought significant challenges across governmental, industrial, and social systems. Additionally, numerous studies have reported a sharp increase in both mental health issues and musculoskeletal disorders worldwide. This study aimed to investigate whether a specific exercise program could alleviate neck and upper back pain symptoms and improve mood state among healthcare and office employees during the post-COVID-19 period. *Materials and Methods*: This was an unblinded (open label) randomized controlled trial (both the participants and the researchers knew which treatment or intervention was being administered). In total, 40 healthcare employees from a public hospital and 98 remote office employees, all of whom reported neck and upper back pain, were randomly assigned to an experimental (EG) and control group (CG). The experimental groups underwent the same exercise protocol, while their corresponding control groups underwent the assessments only. Pain assessment using the Visual Analogue Scale (VAS) and mood state using the Feeling Scale (FS) questionnaires of the participants were recorded at baseline (pre-intervention) and immediately following the exercise intervention period of 6 weeks (post-intervention). The intervention consisted of 18 exercise sessions conducted over a 6-week period, with remote office workers participating online and healthcare workers attending exercise sessions on site (at the hospital). Statistical analysis was performed using non-parametric Mann–Whitney and Wilcoxon tests, as appropriate. *Results*: Following the six-week intervention, the percentage of workers in the EG reporting pain decreased significantly (from 75% to 45% for healthcare workers and from 54% to 25% for office employees), whereas no change was observed for their corresponding control groups. Similarly, the mood state of the EGs significantly improved compared with the control groups, as measured between pre- and post-intervention sessions. *Conclusions*: The COVID-19 period exacerbated stress and musculoskeletal strain, particularly for workers in demanding occupational roles. To mitigate these effects, exercise programs that can be applied while at work should be implemented, as they are effective in enhancing mood and managing neck pain in employees with physical exertion.

## 1. Introduction

The COVID-19 pandemic brought unprecedented changes to workplace environments, particularly for remote office workers and healthcare professionals. Both groups encountered heightened risks of developing work-related musculoskeletal disorders (WMSDs), which have long been recognized as a significant occupational health concern. WMSDs account for more than one-third of occupational injuries, frequently emerging days after the initial strain and contributing to substantial economic costs due to absenteeism and lost productivity. It is estimated that these disorders cost businesses between $15 and $20 billion annually in healthcare expenses and lost work time [1]. The symptoms of WMSDs typically manifest as pain in the muscles, nerves, and joints, particularly in the limbs and spine [2].

For remote office workers, the shift to telecommuting has led to extended periods of sedentary behavior, often compounded by inadequate ergonomic setups in home-based workstations. The association also between prolonged computer use and the development of musculoskeletal disorders is well-established and well-documented [3]. Factors such as poorly designed workstations, inappropriate monitor and keyboard placement, poor posture, and long periods without breaks contribute significantly to the risk of WMSDs [4]. While many of these risks can be mitigated in corporate or public workplaces through structured ergonomic programs, which have been shown to reduce injury rates and improve productivity, job satisfaction, and employee well-being [5,6], responsibility for maintaining proper ergonomics shifts to the individual when working remotely. This transition has exacerbated the musculoskeletal health risks for many employees, as home office environments are often not designed with ergonomics in mind [7,8].

In contrast, healthcare professionals faced a different set of challenges during the pandemic. In addition to the already demanding nature of their work, healthcare employees were subjected to increased physical and mental health risks due to the heightened emotional demands, stricter safety protocols, longer working hours, and heavier workloads brought about by the pandemic [9,10]. Healthcare workers, particularly nurses and surgeons, are already at high risk for WMSDs due to their physically demanding tasks [11,12], which include heavy lifting, repetitive movements, awkward postures, and prolonged standing [13,14]. The prevalence of WMSDs in this population is particularly high, with rates ranging from 28% to 96%, depending on the type of healthcare professional and their specific duties [15].

Given these occupational hazards, numerous intervention strategies have been proposed to alleviate WMSDs in both the office and healthcare settings. Exercise interventions focused on improving stability, flexibility, and posture have been shown to reduce musculoskeletal pain and improve overall physical well-being [16,17,18,19]. These interventions vary in duration and intensity, ranging from brief breaks with posture adjustments to comprehensive exercise programs lasting several weeks [20,21,22,23,24,25]. While such programs have demonstrated moderate success in reducing pain and improving posture, implementing and sustaining these interventions, particularly in remote environments, poses significant challenges [26]. Many workers struggle with self-managing pain in remote settings, often leading to frustration and a cycle of resignation [27,28,29]. This underscores the need for accessible and effective exercise interventions [30] that can be easily integrated into daily routines, both in remote and traditional work environments. It is also particularly relevant, as it addresses a growing need for interventions that can be implemented in the wake of the pandemic, where many employees continue to work remotely or in environments with increased physical and mental demands.

The aim of the present study was to address these challenges by implementing a targeted exercise intervention protocol specifically designed for remote office workers and healthcare professionals at the end of the COVID-19 pandemic. The exercise program was developed to manage and reduce the symptoms experienced by both office workers and healthcare professionals. While office workers and healthcare professionals face different occupational risks, they share common symptoms and ergonomic challenges, particularly in relation to neck pain and upper back discomfort. The intervention sought to evaluate the effectiveness of such an exercise program over 6 weeks in managing neck and upper back symptoms, as well as improving the positive mood of these two occupational populations.

The main hypothesis of this study was that this specific exercise program would significantly reduce self-reported neck and upper back pain among workers in the experimental groups compared with their corresponding control groups after a six-week intervention. A secondary hypothesis was that the exercise program would yield comparable improvements in pain reduction and mood enhancement across different occupational populations (e.g., healthcare workers vs. office workers).

## 2. Materials and Methods

### 2.1. Study Design

This unblinded (open label) randomized controlled trial (both the participants and the researchers knew which treatment or intervention was being administered) was conducted among remote office employees through online sessions and among healthcare employees who attended the exercise intervention on site in a hospital setting. In total, 40 healthcare employees from a public hospital and 98 remote office employees were randomly assigned to an experimental and control group for each occupational population (healthcare and office employees). Both experimental groups underwent the same exercise protocol, while their corresponding control groups participated only in the assessments. Pain assessment and the mood state of the participants were recorded at baseline (pre-intervention) and immediately following the exercise intervention period of 6 weeks (post-intervention).

The same expert who carried out the assessments and guided the intervention was not blinded.

The rationale for dividing healthcare employees and office workers into separate populations and analyzing their results independently lies in the distinct physical and ergonomic demands associated with each occupation. Healthcare employees often perform physically demanding tasks, such as lifting patients, prolonged standing, and repetitive physical movements, which can lead to strain in the neck, back, and shoulders. Conversely, office workers are typically exposed to prolonged sedentary behavior, extended computer use, and suboptimal ergonomic setups, which are associated with musculoskeletal strain in the neck and upper back. Analyzing these populations separately allows for homogeneity and a clearer understanding of how the exercise intervention addresses occupation-specific factors. However, the unequal number of participants across groups, along with the absence of a sample size calculation, represents a limitation of this study.

### 2.2. Inclusion–Exclusion Criteria

This study was announced to a public hospital in northern Greece and to private companies and took place between December 2021 and January 2022. The inclusion criteria required participants to be healthcare or remote office employees, aged 19 to 67, with at least one year of experience in their current position, diagnosed with work-related nonspecific chronic neck and/or upper back pain with the symptoms’ duration being more than 6 months and no prior surgical interventions.

Healthcare employees were defined as those working in hospitals, including surgeons and nurses, considering that nurses and/or nursing assistants are among the most physically active in healthcare, frequently involved in tasks that require strength and stamina. These participants are often in need to lift or reposition patients, move equipment, and spend long hours on their feet. Tasks such as turning and repositioning patients, assisting with mobility, and performing chest compressions during emergencies are also physically demanding and are included in their everyday life.

The participants who were surgeons also often had to stand in one position for hours during surgeries, sometimes maintaining specific postures that require stillness and precision. Many procedures require them to bend over or hold their arms and shoulders in a steady and often tense position for prolonged periods. Their neck and shoulders are particularly prone to tension, as they focus closely on the surgical field.

Office employees were defined as individuals performing solely administrative duties, such as secretaries, clerks, or typists, requiring 8 h of screen time.

Orthopedic doctors who worked in public or private hospitals in Greece provided the diagnosis for all the participants who participated in this study. For the purposes of this study, “nonspecific pain” was defined as pain that is not caused by fractures, direct trauma, or systemic diseases and where no proven root compression exists that would require surgical treatment. The exercise experts and physical therapist who conducted the assessments have more than 20 years of experience in research and evaluations in clinical populations in a university laboratory environment.

### 2.3. Participants

A total of 191 individuals volunteered to participate in this study (88 healthcare employees and 103 office workers). Of these, 138 met the inclusion criteria. The final sample comprised 40 healthcare employees from a public hospital (age range: 45–64 years; males = 1; females = 39) working 40 h per week five days per week, and 98 remote office workers who worked five days per week in eight-hour shifts with screen use (age range: 24–60 years; males = 32; females = 25) without prior exercise experience and medication. No participants dropped out during the course of this study. Informed consent was obtained from all subjects involved in this study. This study was conducted in accordance with the Declaration of Helsinki and approved by the Institutional Ethics Committee of Democritus University of Thrace (15010/133, date of approval 22 October 2024). This study is also registered at the ClinicalTrials.gov Protocol Registration and Results System with identifier NCT06666478.

**Scheme 1:**
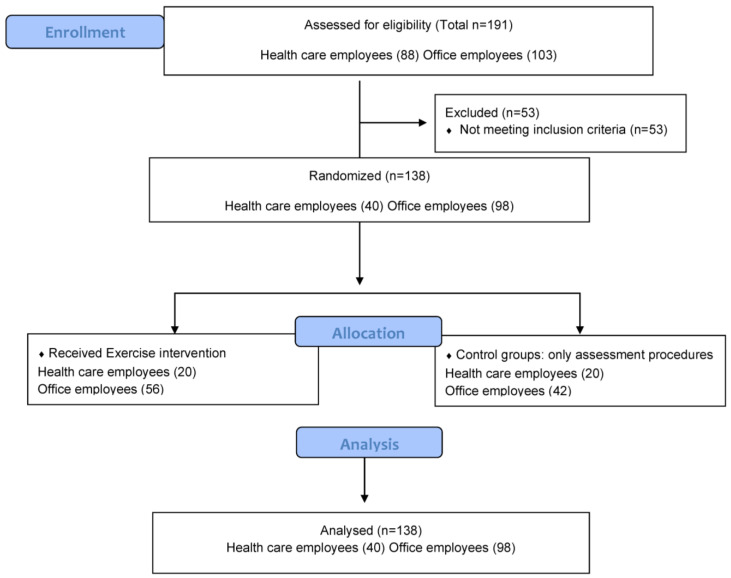
Consort Flow Diagram.

On the first day, all participants (experimental and control groups) were interviewed by the rehabilitation trainer in charge. They also completed two validated questionnaires, which were filled out again post-intervention (i.e., six weeks later) via an online platform. The entire process, including ergonomic training, given to both the experimental and control groups lasted 1.5 h. The ergonomic training content included advice on how to use assistive devices (like mechanical lifts) or apply proper body mechanics and teamwork for healthcare participants. Similarly for office participants in the control group, this training included advice how to adjust their sitting position at home while working, the height of the screen etc. A consort flow diagram is availabe bellow (Scheme 1).

Further details on the measurements are provided below.

### 2.4. Study Variables

#### 2.4.1. Visual Analogue Scale (VAS)

The Visual Analogue Scale (VAS) is a valid, reliable, specific, and sensitive tool for measuring pain and allows patients to rate their pain along a continuum, from “no pain” to “severe pain”, using a 10 cm line. The score is determined by measuring the distance (in mm) between the “no pain” and “severe pain” points, with higher scores indicating greater pain intensity [31].

#### 2.4.2. Feeling Scale (FS)

The Feeling Scale (FS) was used to assess affective valence before and after the intervention. The FS is an 11-point bipolar rating scale that measures current mood on the valence dimension. Odd numbers and zero are verbalized, with the scale ranging from −5 (“very bad”) to +5 (“very good”). The scale was originally developed in English by Hardy and Rejeski [32,33]. The original study introducing the FS established its construct validity by demonstrating that scores on the FS correlated well with physiological markers of exertion and other self-reported measures of affect. One study [32] suggested that the FS is sensitive to changes in affective responses as exercise intensity increases, especially when crossing the lactate threshold, where affect often becomes more negative. The scale was administered in English.

#### 2.4.3. Sociodemographic Variables

Sociodemographic data were collected using an informal questionnaire, which included questions on sex, age, weight, height, occupation, and prior working experience (Table 1).

### 2.5. Intervention

This study was conducted in northern Greece from December 2021 to January 2022 in two experimental phases. The first phase involved remote office employees working five-day eight-hour shifts, while the second phase included healthcare employees working 40 h per week, also for five days per week.

Each occupational group was randomly assigned to an experimental group (EG) or a control group (CG). The exercise intervention for both experimental groups (healthcare and office employees) was designed by the same exercise expert [34], aimed to capture changes in neck and upper back pain, as well as mood, before and after the intervention. The intervention lasted six weeks, with the participants engaging in three 40-min exercise sessions per week. The same expert guided participants through the exercise protocol at the hospital for the healthcare participants and via video calls for the remote office employees. The intervention was administered in groups and not individually. The control groups did not participate in any organized physical activity.

The exercise protocol consisted of mild mobility exercises with fluent movements, strength, stabilization, flexibility, and nerve tissue mobilization, incorporating movements of the upper and lower limbs in both seated and standing positions.

The sessions began in a seated position with spinal mobility exercises, focusing on the hips, lumbar, thoracic, and cervical spine, combined with gradual activation of the upper limb joints. This was followed by multiaxial functional exercises targeting the trunk and upper limbs, mimicking work-related postures such as lateral bending and twisting. Subsequently, the participants performed standing exercises to stretch and mobilize their joints and nerve tissues, promoting relaxation and release. The key points to remember while executing all the exercises were [35] to ensure a proper upright body posture, tilt the pelvis backward, bring the shoulders back and pressed down and finally tuck the chin toward the chest, elongate the head, and breathe normally. Slow execution and flowing movements were emphasized to help the participants develop a stable core during work [35]. Finally, all exercises were performed for a duration of 30 s and with intervals of 20 s between the exercises, always in coordination with breathing, ensuring proper oxygenation of body tissues during inhalation and removal of cellular waste during exhalation [34,35,36].

Regarding nerve tissue mobilization, the following are the basic exercises that can support proper posture and were conducted by the exercise expert in the presence of a physical therapist as well. All these movements were performed in a gentle, controlled manner, with 10 repetitions on each side.

The Median Nerve Glide: While standing or sitting upright with good posture, extend one arm out to the side at shoulder height, palm facing up and slowly extend your wrist and fingers back, so your fingers point toward the floor, tilt your head away from the extended arm to increase the stretch, and then return your head to the center.

The Sciatic Nerve Glide: While sitting on the edge of a chair with both feet flat on the floor, straighten one leg forward, keeping the back straight, flex the foot (toes up toward the shin) and slowly lower the head and chin toward the chest, bring the leg back down, relax the foot, and lift the head. Repeat 10 times on each leg in a slow, controlled motion.

## 3. Statistical Analysis

Statistical analysis was performed using IBM SPSS Statistics for Windows, version 29.0.1. Due to the non-normal distribution of the data, non-parametric statistical methods were employed to assess the significance of differences in pain intensity and mood, both within and between the groups.

The Wilcoxon signed-rank test for paired samples was used to evaluate pre- and post-intervention differences within the same participants in each group. To compare each experimental group (EG) with the corresponding control group (CG), the Mann–Whitney U-test was applied to determine whether there were statistically significant differences between the two groups. A *p*-value of less than 0.05 was considered statistically significant for all analyses.

## 4. Results

### 4.1. Healthcare Employees

**Figure 1:**
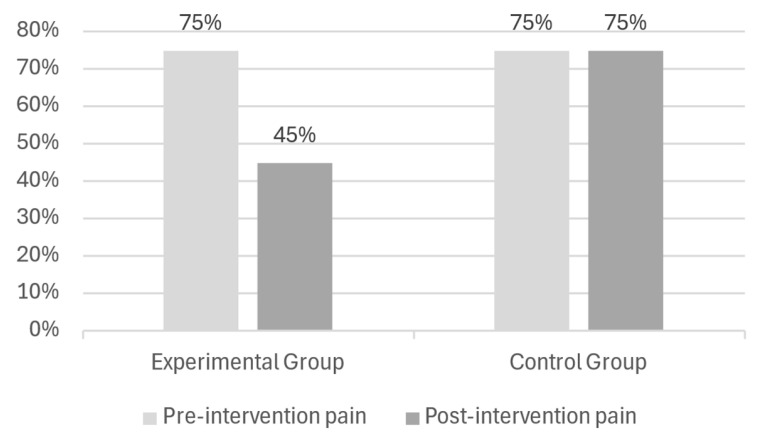
Pre- and post-intervention percentages of healthcare employees reporting neck and upper back pain.

Following the six-week intervention, the percentage of workers in the EG reporting pain decreased from 75% to 45%. In contrast, the CG showed no significant change in the percentage of workers reporting pain (Figure 1).

According to the Wilcoxon signed-rank test, pain intensity in the experimental group was significantly lower post-intervention (median (Md) = 0.0) compared with pre-intervention (Md = 6.0; z = −3.843, *p* < 0.001). Additionally, mood scores were significantly higher post-intervention (Md = 10.0) compared with pre-intervention (Md = 8.0) (Table 2).

The Mann–Whitney U-test revealed that post-intervention pain intensity was significantly lower in the experimental group (EG) (median (Md) = 0.00) compared with the control group (CG) (Md = 6.00; U = 16, z = −5.096, *p* < 0.001), with a large effect size (r = 0.81).

The test also showed a significant difference between the two groups (U = 16, n1 = 20, n2 = 20, *p* < 0.001) with a large effect size (r = 0.81).

Additionally, mood scores were significantly higher in the EG (Md = 10) compared with the CG (Md = 9; U = 124.5, z = −2.120, *p* = 0.034), with a medium effect size (r = 0.33) (Table 3).

### 4.2. Office Employees

Table 4 and Table 5 illustrate the mean values for both groups pre- and post-intervention and for both occupational groups.

**Figure 2:**
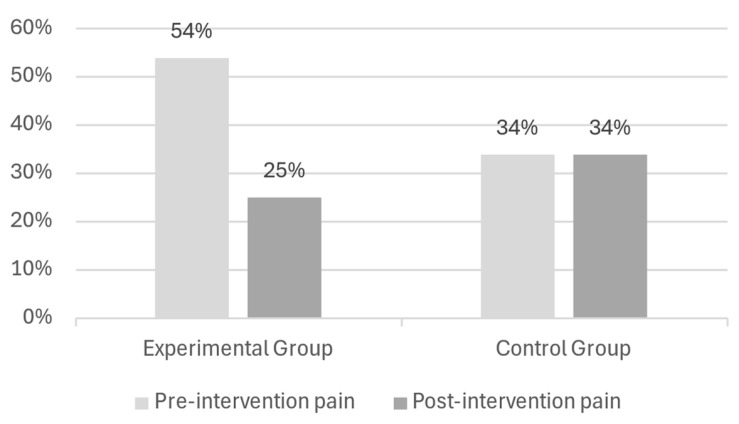
Pre- and post-intervention percentages of office employees reporting neck and upper back pain.

Demographic data for the office employees are shown in Table 1. Concerning the office employees in the experimental group, the percentage of them reporting pain decreased from 54% to 25% after the intervention, whereas no change was observed in the control group (Figure 2).

A Wilcoxon signed-rank test revealed a significant reduction in pain intensity in the experimental group following the intervention. Post-intervention pain levels (median (Md) = 1.0) were significantly lower compared with pre-intervention levels (Md = 5.0; z = −6.434, *p* < 0.001). Additionally, mood scores showed a significant improvement after the intervention, with post-intervention mood (Md = 10.5) being significantly higher than pre-intervention mood (Md = 9.0; z = −5.390, *p* < 0.001) (Table 6).

### 4.3. Pain Intensity

A Mann–Whitney U-test revealed that pain intensity was significantly lower in the experimental group (median (Md) = 0.10, n = 56) compared with the control group (Md = 5.00, n = 42; U = 442, z = −5.386, *p* = 0.001), with a large effect size (r = 0.54). This result indicates that the experimental group experienced significantly lower levels of pain compared with the control group following the intervention (Table 7).

### 4.4. Mood

A Mann–Whitney U-test revealed that mood was significantly higher in the experimental group (median (Md) = 10.0, n = 56) compared with the control group (Md = 7, n = 42; U = 253, z = −6.738, *p* = 0.001), with a large effect size (r = 0.68). This result suggests that the intervention had a significant positive impact on mood in the experimental group compared with the control group (Table 7).

## 5. Discussion

The present study demonstrated that the exercise protocol developed for this research produced significant improvements in neck/upper back pain relief and mood enhancement among both office and healthcare employees. The findings revealed statistically significant differences in the Visual Analogue Scale (VAS) and Feeling Scale (FS) results before and after the intervention for the experimental groups, regardless of occupational background. This suggests that the exercise protocol not only alleviated neck and upper back pain but also positively influenced the mood state of participants, highlighting its efficacy across different work environments with distinct physical demands.

Neck and upper back pain has long been associated with occupational factors, particularly among office employees who are subjected to prolonged sitting and poor ergonomic practices, such as improper neck and shoulder posture. Several studies have previously emphasized the strong link between extended periods of sitting and the development of neck pain in this population [37,38]. Poor posture, particularly a forward head posture and rounded shoulders, is a significant contributor to neck pain and/or upper back pain. Correcting these postural deviations can help alleviate pain and prevent its recurrence [39]. The findings of this study support these assertions, showing a marked reduction in pain following the intervention, particularly in the office employee subgroup, whose work involves extended sedentary tasks that place strain on the upper back and neck.

The office employees in this study demonstrated high needs for stability, flexibility, and mobilization of the upper back and neck regions. The exercise protocol was designed with these specific requirements in mind, providing targeted mobility exercises that progressively engaged the spinal and peripheral joints, addressing the postural and movement limitations that commonly affect office workers. By incorporating exercises that simulate typical office work postures (such as forward head posture and rounded shoulders), the intervention allowed the participants to improve their posture and reduce tension in key areas prone to pain. These improvements are likely to reduce the cumulative physical strain office employees experience due to static postures and repetitive movements, which are common in this occupational setting.

In contrast, healthcare employees face different physical demands, as their work often requires a combination of physical strength, coordination, and endurance. The exercise program for healthcare employees was therefore adapted to focus on enhancing stability, strength, flexibility, and coordination while standing, which are essential for their daily tasks. Healthcare workers, such as nurses and surgeons, are frequently exposed to dynamic work environments that involve lifting, bending, and other physically strenuous activities. Consequently, the intervention incorporated functional exercises that promote spinal stabilization and postural control, both of which are critical for preventing NP in physically demanding jobs.

The literature emphasizes the importance of postural education while standing or bending and frequent corrections to maintain an upright, neutral posture throughout the workday [40]. In particular, the motor control system for posture plays a key role in ensuring that proper alignment becomes habitual, helping to mitigate the long-term consequences of improper posture. This principle was integrated into the intervention program, which encouraged participants to make conscious corrections to their posture during the day, reinforcing healthier movement patterns over time.

Muscle-strengthening and muscle endurance exercises have also proven essential for addressing postural imbalances that can lead to neck and upper back pain. Poor posture, particularly a forward head posture, places excessive strain on the cervical spine, altering muscle length and leading to discomfort or chronic pain in the upper back [41]. Strengthening the deep neck flexors and the muscles of the upper back is crucial for restoring neuromuscular balance and stabilizing the cervical spine. The intervention exercises highlighted in this study were specifically designed to target these muscle groups, promoting improved posture and muscle function with many repetitions and a duration of 20 to 30 s for activation. Prior studies have demonstrated the effectiveness of strengthening and muscle endurance training in reducing pain and improving postural alignment [37,42], which aligns with the outcomes observed in this study.

Furthermore, the positive effects of the exercise protocol extended beyond physical pain relief, as demonstrated by the significant improvements in the participants’ mood state. Mood enhancement may be linked to several factors, including the reduction in physical discomfort, improved mobility, and the psychological benefits associated with physical activity. The structured nature of the exercise sessions, which incorporated coordination, flowing movements, and rhythmic breathing, likely contributed to a more relaxed and focused state, resulting in a better mood post-intervention. This highlights the holistic impact of this exercise program that not only addresses physical discomfort but also promotes mental health and overall well-being.

These findings might be related to the nature of the exercise protocol designed. Its primary advantage is its ease of integration into daily routines, allowing workers to perform the exercises while seated or standing without drawing attention to themselves. The protocol requires no special equipment and can be carried out in regular office attire, making it highly accessible. Additionally, the exercises are simple to adopt following initial instruction from the exercise expert. After minimal practice, the participants were able to monitor their own execution and maintain proper posture while working [43].

The most common methodological problem that authors must face usually is to collect patients with the same or similar diagnoses. The same limitation has been addressed by the authors of the present study using the IASP’s basic criterion for the definition of pain based on the duration of symptoms. The IASP recognizes chronic pain in general as any pain that has persisted for longer than three months, although, for research purposes, it prefers six months as the defining period.

Another limitation of this study was that the demographic data did not include any information on mental illness e.g., depression, mental illness, or bipolar disorder.

Despite these limitations, the results of this study provide strong evidence for the effectiveness of the exercise protocol in reducing neck and upper back pain and improving mood in both office and healthcare employees. By tailoring the intervention to the specific needs of each occupational group, the program successfully addressed the different physical demands faced by the participants. The incorporation of spine mobility exercises, stability, endurance strengthening, and postural correction exercises made the protocol highly relevant to the participants’ work environments, demonstrating its potential as a practical and accessible intervention for managing musculoskeletal problems and enhancing mood in diverse work settings.

The findings of this study support the generalizability of the exercise protocol across similar occupational groups experiencing work-related musculoskeletal issues. By addressing both office and healthcare employees—two populations with distinct ergonomic and physical demands—this study demonstrates that the intervention’s core components (spine mobility exercises, stability and endurance strengthening, and postural correction) are adaptable to diverse work settings. This adaptability enhances the protocol’s potential applicability in other occupational environments with similar musculoskeletal strain, such as education, retail, and manufacturing, where workers also face prolonged sedentary periods or repetitive physical tasks.

Moreover, the use of non-specialized equipment and the ability to perform the exercises in standard work attire make the protocol practical for broader implementation. However, while the results indicate effectiveness within the healthcare and office settings, further research should explore additional occupational groups and work contexts to confirm the intervention’s applicability across varied job roles. It would also be valuable to conduct studies in different cultural or geographical settings to assess any environmental or lifestyle factors that could influence the outcomes.

In summary, this study provides promising evidence for the exercise protocol’s effectiveness in reducing neck and upper back pain and improving mood. Although further research is needed to establish its broader applicability, the protocol shows strong potential as a practical, accessible solution for managing musculoskeletal health in diverse occupational environments.

## 6. Conclusions

The present study demonstrates that the exercise intervention, designed with specific characteristics aimed at improving stability and flexibility, had a significant effect on reducing neck and upper back pain and enhancing the musculoskeletal health of the upper back—areas frequently affected in various occupational populations. The intervention specifically targeted muscle stability and flexibility in the upper back, which are crucial in mitigating the negative effects of repetitive movements, prolonged static postures, and poor ergonomic conditions in the workplace.

The findings underscore the importance of regular mobility exercises to counteract the strain caused by occupational tasks, particularly in the post-COVID-19 era, when remote work and sedentary lifestyles have become more prevalent. Poor ergonomics and long working hours in fixed positions, such as sitting at a desk or performing repetitive tasks in healthcare settings, are well-known contributors to work-related musculoskeletal disorders (WMSDs). The intervention protocol demonstrated that it is possible to address these issues effectively with structured, short-duration exercise sessions, even when conducted remotely.

A key challenge moving forward is ensuring the widespread adoption and sustainability of such interventions in the workplace. Implementing this exercise program in a larger number of workplaces could contribute to long-term reductions in WMSDs and promote healthier, more ergonomic work practices. Furthermore, encouraging workers to integrate these exercises into their daily routines and maintain them as lifelong habits would not only alleviate acute pain but also prevent future musculoskeletal issues.

Future efforts should focus on integrating this type of intervention within occupational health programs and exploring its scalability across diverse work environments. Additionally, further research is needed to assess the long-term benefits of these interventions and to investigate strategies for enhancing workers’ adherence to these exercise routines. Overall, this study highlights the potential of targeted exercise interventions as a cost-effective and impactful solution for improving workplace health and reducing the burden of WMSDs.

## Figures and Tables

**Table 1:** Sociodemographic data for the experimental and control groups of participants.

Sociodemographic	Healthcare Employees (N = 40)		Office Employees (N = 98)	
	EG (N = 20)	CG (N = 20)	EG (N = 56)	CG (N = 42)
	Μ ± SD	Μ ± SD	Μ ± SD	Μ ± SD
Age	55.10 ± 5.67	54.62 ± 3.04	53.6 ± 3.58	50.70 ± 2.04
Height	163.75 ± 6.68	164.85 ± 4.69	168.62 ± 7.01	165.54 ± 3.69
Weight (kg)	70.35 ± 7.75	70.10 ± 3.87	68.22 ± 7.90	66.06 ± 4.01
Years of work	4.00 ± 0.00	3.80 ± 0.41	5.00 ± 0.00	3.65 ± 0.41

**Table 2:** Pre- and post-intervention pain intensity (VAS) and mood state (FS) in the experimental group of healthcare employees.

	Pre		Post		Z	Wilcoxon Signed-Rank Test Probability
	**Md**	**SD**	**Md**	**SD**		
Pain intensity	6.00	1.932	0.00	1.673	−3.843	<0.001
Mood state	8.00	2.328	10.00	1.234	−3.438	<0.001

**Table 3:** Differences between experimental and control group of healthcare employees.

Categories	Group	N	Median	U	Z	*p*
Pain intensity	EG	20	0.0	16.0	−5.096	<0.001
	CG	20	6.0			
Mood State	EG	20	10.0	124.5	−2.120	0.034
	CG	20	9.0			

**Table 4:** Mean values and standard deviations (Μ ± SD) of pain intensity for all the groups, pre- and post-intervention.

Pain Intensity	Healthcare Employees (N = 40)		Office Employees (N = 98)	
	Pre	Post	Pre	Post
Groups	Μ ± SD	Μ ± SD	Μ ± SD	Μ ± SD
Experimental group	6.05 ± 1.93	1.20 ± 1.67	4.79 ± 1.53	2.50 ± 0.3
Control group	6.15 ± 1.87	6.11 ± 1.13	6.15 ± 1.87	6.15 ± 1.87

**Table 5:** Mean values and standard deviations (Μ ± SD) of mood state for all the groups, pre- and post-intervention.

Mood State	Healthcare Employees (N = 40)		Office Employees (N = 98)	
	Pre	Post	Pre	Post
Groups	Μ ± SD	Μ ± SD	Μ ± SD	Μ ± SD
Experimental group	8.05 ± 2.32	10.05 ± 1.23	7.50 ± 1.53	9.50 ± 0.3
Control group	8.80 ± 1.98	8.80 ± 1.98	7.95 ± 1.87	7.88 ± 1.87

**Table 6:** Pre- and post-intervention pain intensity (VAS) and mood state (FS) in the experimental group of office employees.

	Before		After		*Z*	Wilcoxon Signed-Rank Test Probability
	Md	SD	Md	SD		
Pain intensity	5.00	2.193	1.00	1.888	−6.434	<0.001
Mood state	9.00	2.106	10.50	1.470	−5.390	<0.001

**Table 7:** Differences between experimental and control group of office employees.

Categories	Group	N	Median	U	Z	*p*
Pain intensity	EG	56	0.10	442	−5.386	0.001
	CG	42	5.0			
Mood state	EG	56	10.0	253	−6.738	0.001
	CG	42	7.0			

## Data Availability

Data are unavailable due to privacy restrictions.

## References

[1] Koma B.S., Bergh A.-M., Costa-Black K.M. (2019). Barriers to and facilitators for implementing an office ergonomics programme in a South African research organisation. Appl. Ergon..

[2] Buomprisco G., Ricci S., Perri R., De Sio S. (2021). Health and Telework: New Challenges after COVID-19 Pandemic. Eur. J. Environ. Public Health.

[3] de Macêdo T.A.M., Cabral E.L.d.S., Silva Castro W.R., de Souza Junior C.C., da Costa Junior J.F., Pedrosa F.M., da Silva A.B., de Medeiros V.R.F., de Souza R.P., Cabral M.A.L. (2020). Ergonomics and telework: A systematic review. Work.

[4] Janneck M., Jent S., Weber P., Nissen H. (2017). Ergonomics To Go: Designing The Mobile Workspace. Int. J. Hum. Comput. Interact..

[5] Fischer C., Siegel J., Proeller I., Drathschmidt N. (2023). Resilience through digitalisation: How individual and organisational resources affect public employees working from home during the COVID-19 pandemic. Public Manag. Rev..

[6] Wütschert M.S., Romano-Pereira D., Suter L., Schulze H., Elfering A. (2022). A systematic review of working conditions and occupational health in home office. Work.

[7] MacEwen B.T., MacDonald D.J., Burr J.F. (2015). A systematic review of standing and treadmill desks in the workplace. Prev. Med..

[8] Ráthonyi G., Kósa K., Bács Z., Ráthonyi-Ódor K., Füzesi I., Lengyel P., Bába B. (2021). Changes in workers’ physical activity and sedentary behavior during the COVID-19 pandemic. Sustainability.

[9] Liu Z., Shi Y., Yang B. (2022). Open Innovation in Times of Crisis: An Overview of the Healthcare Sector in Response to the COVID-19 Pandemic. J. Open Innov. Technol. Mark. Complex..

[10] Miranda F.M.D., Santana L.D.L., Pizzolato A.C., Saquis L.M.M. (2020). Working conditions and the impact on the health of the nursing professionals in the context of covid-19. Cogitare Enferm..

[11] Kgakge K., Hlongwa M., Ginindza T. (2021). The distribution of work-related musculoskeletal disorders among nurses in sub-Saharan Africa: A scoping review protocol. Syst. Rev..

[12] Munabi I.G., Buwembo W., Kitara D.L., Ochieng J., Mwaka E.S. (2014). Musculoskeletal disorder risk factors among nursing professionals in low resource settings: A cross-sectional study in Uganda. BMC Nurs..

[13] Albanesi B., Piredda M., Bravi M., Bressi F., Gualandi R., Marchetti A., Facchinetti G., Ianni A., Cordella F., Zollo L. (2022). Interventions to prevent and reduce work-related musculoskeletal injuries and pain among healthcare professionals. A comprehensive systematic review of the literature. J. Saf. Res..

[14] Ateş R., Yakut H. (2023). Investigation of musculoskeletal disorders, physical activity level, sleep quality, and fatigue in health professionals with and without a history of COVID-19. Work.

[15] Aghilinejad M., Ehsani A.A., Talebi A., Koohpayehzadeh J., Dehghan N. Ergonomic Risk Factors and Musculoskeletal Symptoms in Surgeons with Three Types of Surgery: Open, Laparoscopic, and Microsurgery. http://mjiri.iums.ac.ir.

[16] Sheahan P.J., Diesbourg T.L., Fischer S.L. (2016). The effect of rest break schedule on acute low back pain development in pain and non-pain developers during seated work. Appl. Ergon..

[17] Gustafsson K., Marklund S. (2011). Consequences of Sickness Presence and Sickness Absence on Health and Work Ability: A Swedish Prospective Cohort Study. Int. J. Occup. Med. Environ. Health.

[18] Ferreira E.J., Strydom E.A. (2016). Managing work-related musculoskeletal disorders in the virtual office. J. Contemp. Manag..

[19] Parry S.P., Coenen P., Shrestha N., O’Sullivan P.B., Maher C.G., Straker L.M. (2019). Workplace interventions for increasing standing or walking for decreasing musculoskeletal symptoms in sedentary workers. Cochrane Database Syst. Rev..

[20] Shariat A., Cleland J.A., Danaee M., Kargarfard M., Sangelaji B., Tamrin S.B.M. (2018). Effects of stretching exercise training and ergonomic modifications on musculoskeletal discomforts of office workers: A randomized controlled trial. Braz. J. Phys. Ther..

[21] Marins E.F., Caputo E.L., Krüger V.L., Junior D.M., Scaglioni F.G., Del Vecchio F.B., Primo T.T., Alberton C.L. (2023). Effectiveness of m-health-based core strengthening exercise and health education for public safety workers with chronic non-specific low back pain: Study protocol for a superiority randomized controlled trial (SAFEBACK). Trials.

[22] Garcia M.G., Estrella M., Peñafiel A., Arauz P.G., Martin B.J. (2023). Impact of 10-Min Daily Yoga Exercises on Physical and Mental Discomfort of Home-Office Workers During COVID-19. Hum. Factors.

[23] Mehta S., Chahal A., Malik S., Rai R.H., Malhotra N., Vajrala K.R., Sidiq M., Sharma A., Sharma N., Kashoo F.Z. (2024). Evading Musculoskeletal Conditions Using Qigong as a Rescue Technique. J. Lifestyle Med..

[24] Vitoulas S., Konstantis V., Drizi I., Vrouva S., Koumantakis G.A., Sakellari V. (2022). The Effect of Physiotherapy Interventions in the Workplace through Active Micro-Break Activities for Employees with Standing and Sedentary Work. Healthcare.

[25] Parés-Salomón I., Señé-Mir A.M., Martín-Bozas F., Loef B., Coffey A., Dowd K.P., Jabardo-Camprubí G., Proper K.I., Puig-Ribera A., Bort-Roig J. (2024). Effectiveness of workplace interventions with digital elements to reduce sedentary behaviours in office employees: A systematic review and meta-analysis. Int. J. Behav. Nutr. Phys. Act..

[26] Lavé A., Gondar R., Demetriades A.K., Meling T.R. (2020). Ergonomics and musculoskeletal disorders in neurosurgery: A systematic review. Acta Neurochir..

[27] Argent R., Daly A., Caulfield B. (2018). Patient involvement with home-based exercise programs: Can connected health interventions influence adherence?. JMIR Mhealth Uhealth.

[28] Brusaca L.A., Barbieri D.F., Mathiassen S.E., Holtermann A., Oliveira A.B. (2021). Physical behaviours in brazilian office workers working from home during the COVID-19 pandemic, compared to before the pandemic: A compositional data analysis. Int. J. Environ. Res. Public Health.

[29] Aegerter A.M., Deforth M., Johnston V., Sjøgaard G., Volken T., Luomajoki H., Dratva J., Dressel H., Distler O., Elfering A. (2021). No evidence for an effect of working from home on neck pain and neck disability among Swiss office workers: Short-term impact of COVID-19. Eur. Spine J..

[30] Robelski S., Keller H., Harth V., Mache S. (2019). Coworking spaces: The better home office? A psychosocial and health-related perspective on an emerging work environment. Int. J. Environ. Res. Public Health.

[31] Delgado D.A., Lambert B.S., Boutris N., McCulloch P.C., Robbins A.B., Moreno M.R., Harris J.D. (2018). Validation of Digital Visual Analog Scale Pain Scoring with a Traditional Paper-based Visual Analog Scale in Adults. J. Am. Acad. Orthop. Surg. Glob. Res. Rev..

[32] Thorenz K., Berwinkel A., Weigelt M. (2023). A Validation Study for the German Versions of the Feeling Scale and the Felt Arousal Scale for a Progressive Muscle Relaxation Exercise. Behav. Sci..

[33] Hardy C.J., Rejeski W.J. (1989). Not What, but How One Feels: The Measurement of Affect during Exercise. J. Sport Exerc. Psychol..

[34] Beneka A. Exercise at workspace as a snack prevents muscleskeletal problems and promotes workers’ health. Proceedings of the Balkan Health and Safety at Work Congress 2024.

[35] Amiri B., Zemková E. (2023). Diaphragmatic breathing exercises in recovery from fatigue-induced changes in spinal mobility and postural stability: A study protocol. Front. Physiol..

[36] Pleil J.D., Wallace M.A.G., Davis M.D., Matty C.M. (2021). The physics of human breathing: Flow, timing, volume, and pressure parameters for normal, on-demand, and ventilator respiration. J. Breath Res..

[37] Falla D., Jull G., Russell T., Vicenzino B., Hodges P. (2007). Effect of neck exercise on sitting posture in patients with chronic neck pain. Phys. Ther..

[38] Chung Y., Lee S., Lee Y. (2017). Effect of changes in head postures during use of laptops on muscle activity of the neck and trunk. Phys. Ther. Rehabil. Sci..

[39] Thigpen C.A., Lynch S.S., Mihalik J.P., Prentice W.E., Padua D. (2010). The effects of an exercise intervention on forward head and rounded shoulder postures in elite swimmers. Br. J. Sports Med..

[40] Abdollahzade Z., Shadmehr A., Malmir K., Ghotbi N. (2017). Effects of 4 Week Postural Corrective Exercise on Correcting Forward Head Posture. J. Mod. Rehabil..

[41] Gram B., Holtermann A., Bültmann U., Sjøgaard G., Søgaard K. (2012). Does an Exercise Intervention Improving Aerobic Capacity Among Construction Workers Also Improve Musculoskeletal Pain, Work Ability, Productivity, Perceived Physical Exertion, and Sick Leave?: A Randomized Controlled Trial. J. Occup. Environ. Med..

[42] Lee J., Kim D., Yu K., Cho Y., You J.H. (2018). Comparison of isometric cervical flexor and isometric cervical extensor system exercises on patients with neuromuscular imbalance and cervical crossed syndrome associated forward head posture. Biomed. Mater. Eng..

[43] Beneka A., Malliou P., Gioftsidou A. (2014). Neck Pain and Office Workers: An Exercise Program for the Workplace. ACSM’s Health Fit. J..

